# Supplementary material for: Impact of Formulation Conditions on Lipid Nanoparticle Characteristics and Functional Delivery of CRISPR RNP for Gene Knock-Out and Correction
Source: Pharmaceutics. 2022 Jan 17;14(1):213. doi: 10.3390/pharmaceutics14010213 (PMC8778360; doi:10.3390/pharmaceutics14010213)
Supplement: Supplementary file 1 [file pharmaceutics-14-00213-s001.zip › pharmaceutics-1524798-supplementary.pdf]

pharmaceutics

Supplementary Materials

Impact of Formulation Conditions on Lipid Nanoparticle Characteristics and Functional Delivery of CRISPR RNP for Gene Knock-Out and Correction

Johanna Walther †, Danny Wilbie †, Vincent S. J. Tissingh, Mert Öktem, Heleen van der Veen, Bo Lou and Enrico Mastrobattista *

Department of Pharmaceutics, Utrecht Institute of Pharmaceutical Sciences (UIPS), Utrecht University, The Netherlands; j.walther@uu.nl (J.W.); d.wilbie@uu.nl (D.W.); vincenttissingh@gmail.com (V.S.J.T.); m.oktem@uu.nl (M.Ö.); h.vanderveen@students.uu.nl (H.v.d.V.); bo.lou@outlook.com (B.L.)

* Correspondence: e.mastrobattista@uu.nl

† These authors contributed equally to this work.

Synthetic genetic sequences used in this work

Table S1. Guide RNA spacer sequences used in this work.

Target	20 nt Spacer Sequence
Stoplight construct	GGACAGUACUCCGCUCGAGU
EGFP construct	GCUGAAGCACUGCACGCCGU

Table S2. PCR primers used for amplification of the Stoplight and EGFP loci, for T7E1 and TIDE as specified in the primer code.

Primer Code	Sequence 5′–3′
Stoplight (T7E1) Forward	GAAGGGCGAGATCAAGCAGA
Stoplight (T7E1) Reverse	GGTCTTGTAGTTGCCGTCGT
Stoplight (TIDE) Forward	GGACGGCGAGTTCATCTACA
Stoplight (TIDE) Reverse	CTTCATGTGGTCGGGGTAGC
EGFP (T7E1) Forward	CGTAAACGGCCACAAGTTCA
EGFP (T7E1) Reverse	GTCCATGCCGAGAGTGATCC

Table S3. Template DNA used in the EGFP to BFP mutation assay. DNA mismatches, encoding the mutation, are highlighted blue. The PAM sequence, needed for Cas9 activity, is additionally mutated in this sequence.

**86 bp (40bp Homology arms)**	**CAAGCTGCCCGTGCCCTGGCCCACCCTCGTGACCACCCTGAGCCACGGCGTGCAGTGCTTCAGCCGCTACCCCGACCACATGAAGC**

Model validation using BFP-expressing plasmid transfection

The designed BFP-expressing gene was ordered in a pET17 vector from Twist Biosience. The full plasmid map is given below in figure S.1. This gene was transfected into HEK293T cells in a 6-well plate using Lipofectamine CRISPRMax (Thermo Scientific) using the manufacturer's specifications. Cells were grown for 2 days and harvested by trypsinization. Flow cytometry was performed to assess the signal and separation of the signal compared to HEK293T-EGFP cells. Both samples were acquired separately, as well as a mixed sample, to optimize the machine settings and separate the signals.

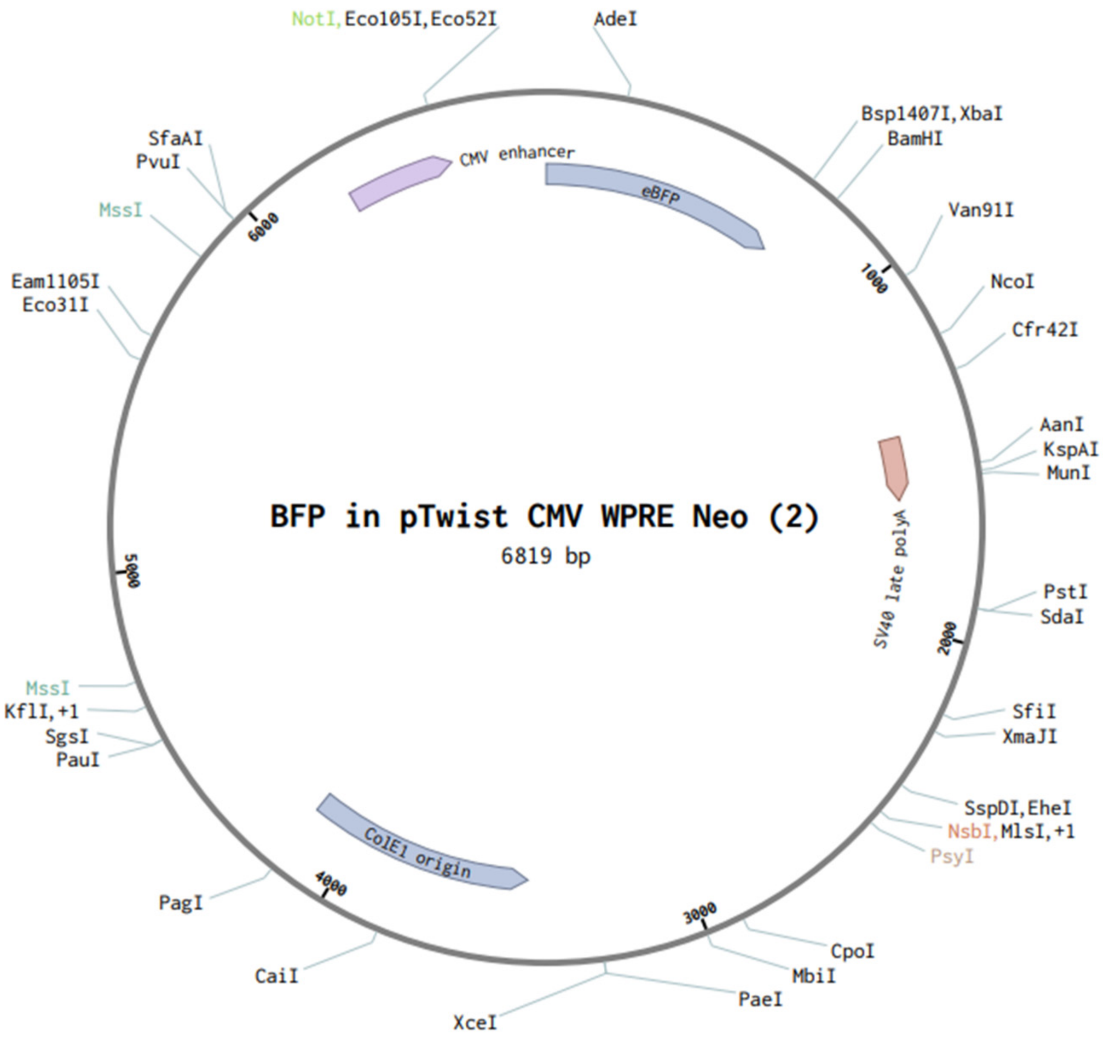

Figure S1. Plasmid map encoding the mutant EGFP gene, which encodes a blue fluorescent protein.

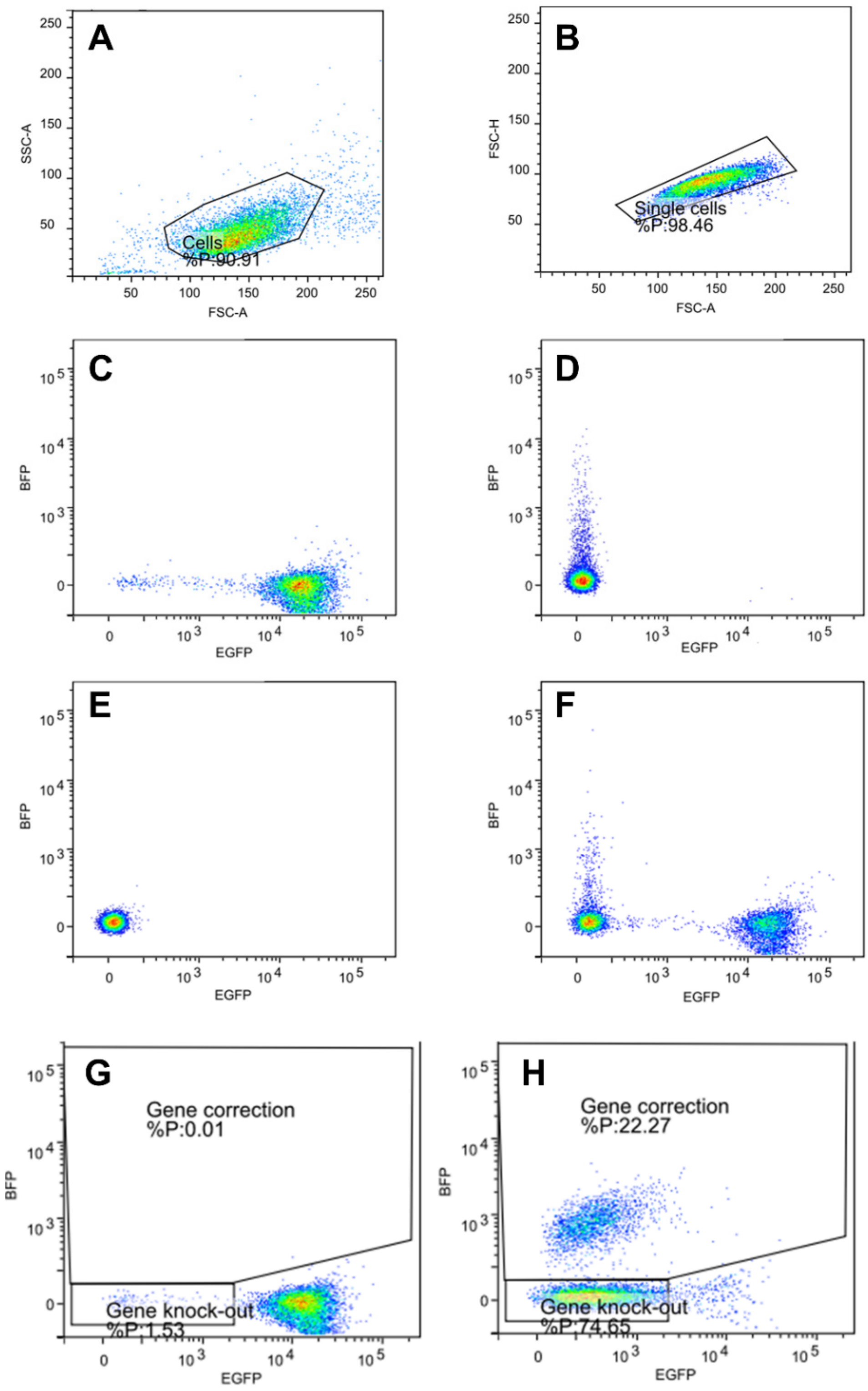

Figure S2. A and B: gating strategy to find single cells employed in all flow cytometry experiments using HEK293T cells. C: HEK293T cells expressing EGFP. D: HEK293T cells transfected with pTwist_BFP. E: HEK293T cells. F: mixed population of C and D to assess the ability to distinguish BFP and EGPF signals. G: untreated HEK-EGFP cells from the dose-escalation study presented in Fig. 6 of the main text. H: LNP-RNP-HDR at 0.25% DOTAP, a 1:2 molar ratio of RNP to template DNA and concentration of 30nM of RNP in the well, after formulation in nuclease free water.

Additional information regarding the stability study of SpCas9 protein

The linear plasmid used is 10,930 base pairs long. After cleavage of the EGFP site, two strands of 7926 and 3004 base pairs are formed. The gels were analyzed by densitometry in ImageJ to calculate the cleaving efficiency of the SpCas9 protein. A background subtraction was performed using a rolling ball radius of 50 pixels. Lanes were drawn in the middle of the bands. The areas of each peak were calculated (AUC) and the activity was calculated by dividing the AUC of the digested bands by the AUC of the sum of all bands.

The purity of AF647-Cas9 was determined using SDS-PAGE and measured by fluorescence using the UV/Stain free/Blot free sample tray and the Alexa 647 preset on the Chemidoc imager. Here it is notable that a small fluorescent population is visible under the front of the loading dye, which may correspond to the free AF647 label. This is also seen in the final sample, which contained the crude labeled protein prior to purification. The Cas9 additionally shows up as a fluorescent band high in the gel around 160 kDa, which is expected. The peak area of the free dye (under the front) was approximately 1% as determined by densitometry. Most of the impurities were found to be larger than the original Cas9 molecular weight, which is in contrast to SDS-PAGE of the unlabeled protein as seen in Fig. 1b.

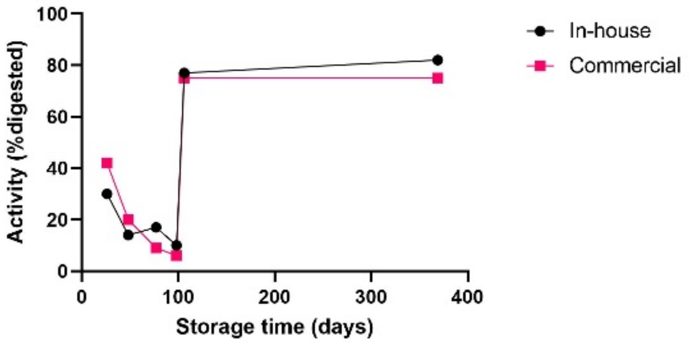

Figure S3. Calculated in vitro SpCas9 activity calculated over a long storage time. Variation between assays is thought to be due to plasmid quality, which is why the protein was compared to a commercially available control each time.

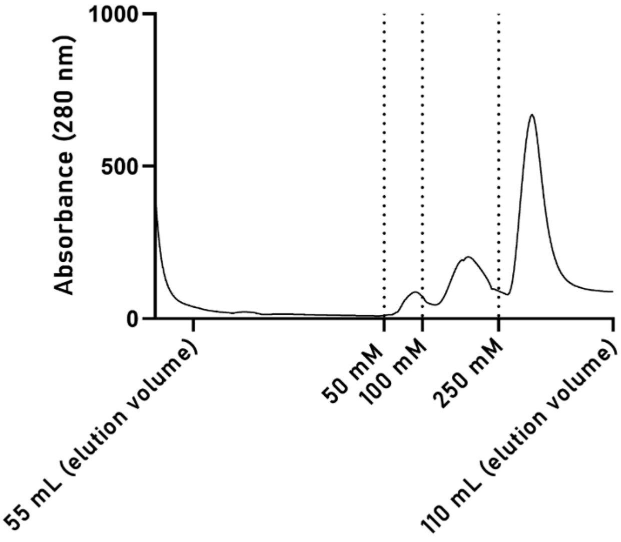

Figure S4. Elution chromatogram of SpCas9 during His-tag purification.

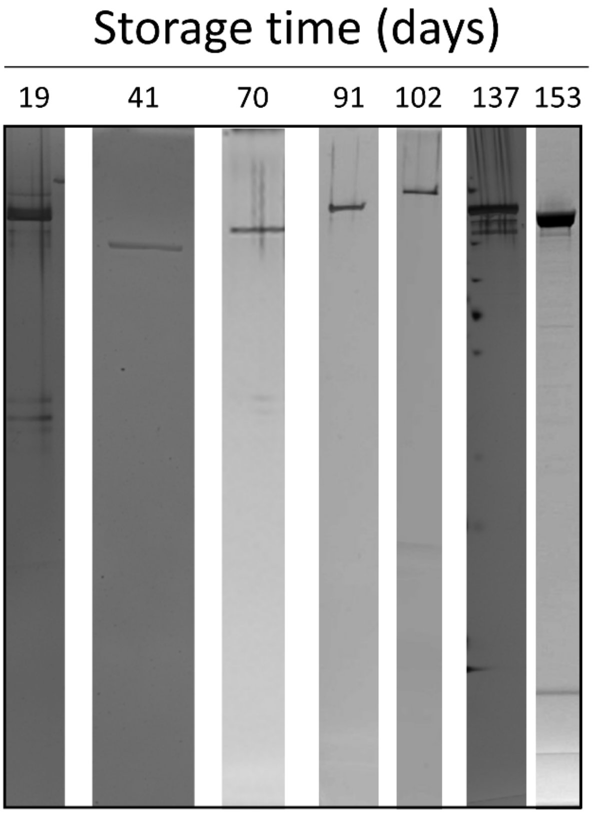

Figure S5.: SDS-PAGE gel excerpt used in the gel densitometry stability study [1]

[1]: Alonso Villela, S.M.; Kraïem, H.; Bouhaouala-Zahar, B.; Bideaux, C.; Aceves Lara, C.A.; Fillaudeau, L. A Protocol for Recombinant Protein Quantification by Densitometry. *MicrobiologyOpen* **2020**, *9*, 1175–1182, doi:10.1002/mbo3.1027.

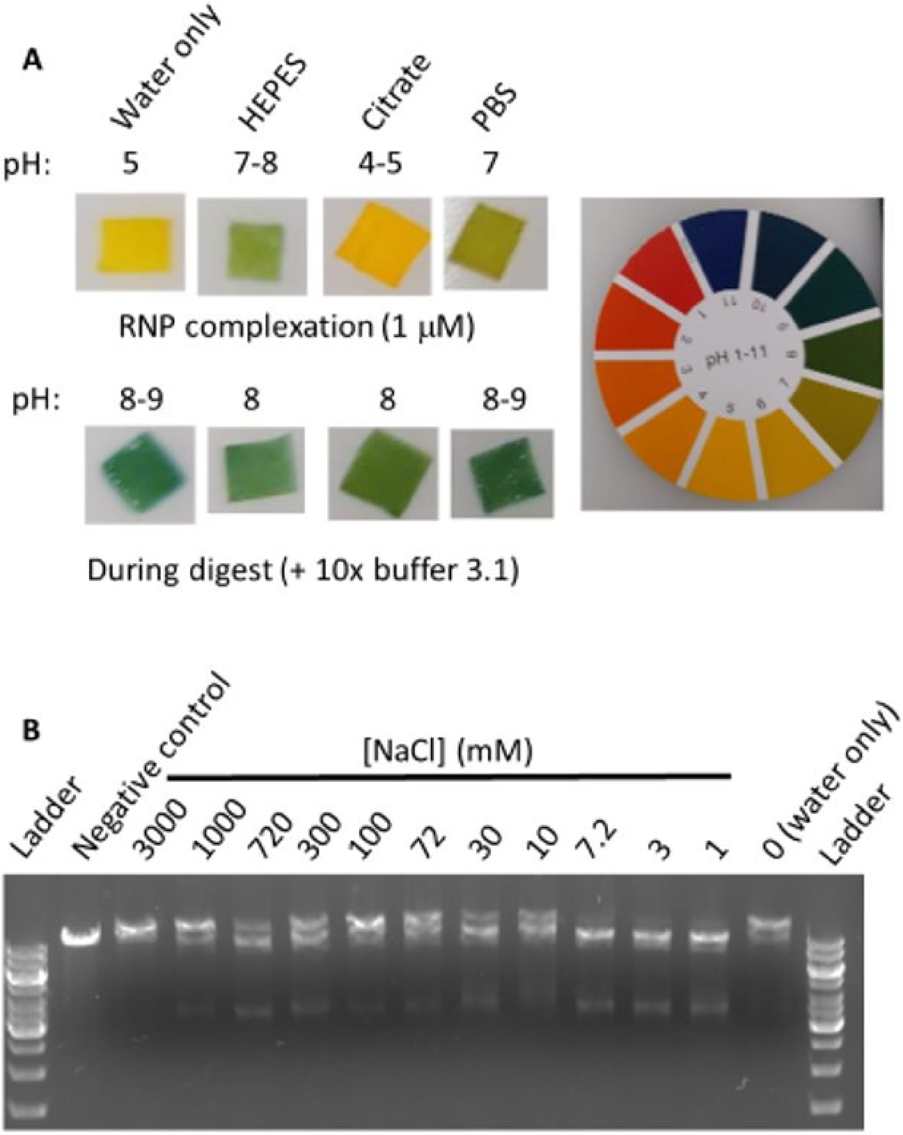

Figure S6.*:* In vitro cleavage activity assay of RNP complexed in various conditions as used during nanopaticle formulation. (**A**) pH values measured with pH paper of the different conditions. (**B**) Agarose gel of the in vitro cleavage activity assay with various sodium chloride concentrations.

Additional information regarding lipid nanoparticle formulation

Table S4.: Schematic representation of LNP preparation with the exact volumes for an exemplary LNP-RNP formulation.

**100 µL LNP formulation; c(RNP) = 0.4 µM; c(total lipids) = 2.2 mM; 50 mM HEPES buffer pH 7.4; 5 % DOTAP**	
**Material**	
1. RNP complexation	
20 µM sgRNA	0.36
3.75 µM SpCas9	1.92
*HEPES buffer was added to both gRNA and SpCas9 to a final volume of 9 µL, respectively<br>i. add 9 µL of 0.8 µM SpCas9 to 9 Ll of 0.8 µM sgRNA<br>ii. incubate for 15 min at RT	
2. Preparation of lipid mixture	
20 mM C12-200	0.23
10 mM DOPE	0.21
10 mM cholesterol	0.61
1 mM PEG-DMG	0.33
7.12 mM DOTAP*	0.09
* EtOH was added to lipid mixture to a total volume of 6 µL	
3. LNP formation	
i. add 25.44 µL of RNP to 6 µl of lipids<br>ii. incubate for 15 min at RT<br>iii. dilute 4× with 1× PBS to final formulation volume of 100 µL	

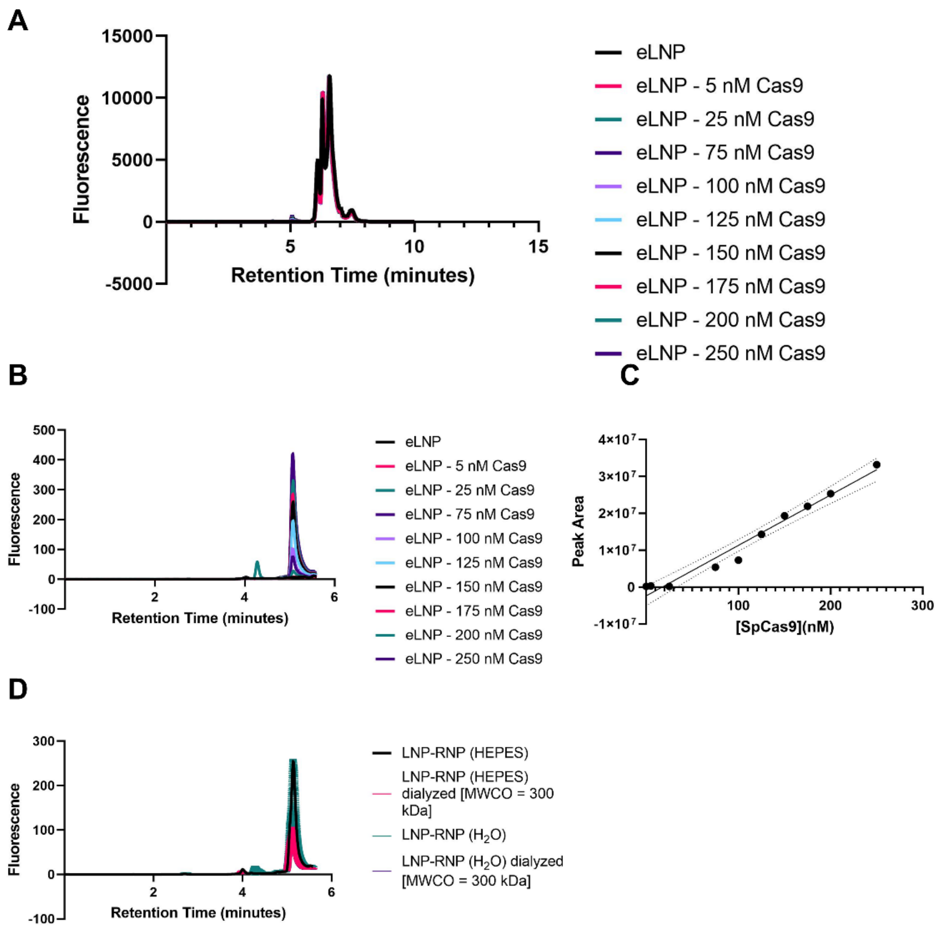

E

	LNP-RNP dialyzed [HEPES]	LNP-RNP [HEPES]	LNP-RNP dialyzed [H_2O]	LNP-RNP [H_2O]
[SpCas9](nM)	*104.82*	164.54	*119.6*	163.57
Complexation efficiency	**63.7 %**		**73.2 %**	

Figure S7.: Determination of complexation efficiency of SpCas9 in LNP. (**A**) Chromatogram (fluorescent detector; ex. 280 nm, em. 350 nm) of the full HPLC run on the Xbridge protein BEH C4 300Å column of empty LNP spiked with different SpCas9 concentrations for calibration. (**B**) Zoomed-in chromatogram of the SpCas9 peak in samples of empty LNP with different SpCas9 concentrations for calibration. (**C**) Calibration curve determined with EMPOWER software. (Linear fit equation: $y = 1.35 \times 10^5 x - 1.94 \times 10^6$; $R^2 = 0.970$) (**D**) Chromatogram of LNP-RNP where the RNP was formulated in 50 mM HEPES pH 7.35 or nuclease-free water (non-dialyzed vs dialyzed) and with 5 mole% DOTAP. (**E**) Overview of the determined molar concentration of SpCas9 in the LNP samples. Complexation efficiency was determined by dividing the concentration of SpCas9 of the dialyzed samples by the non-dialyzed sample, respectively for the two different RNP conditions. Dilution factor of 1.3 was included in the calculations as samples were slightly diluted during dialysis (indicated by italic numbers).

A

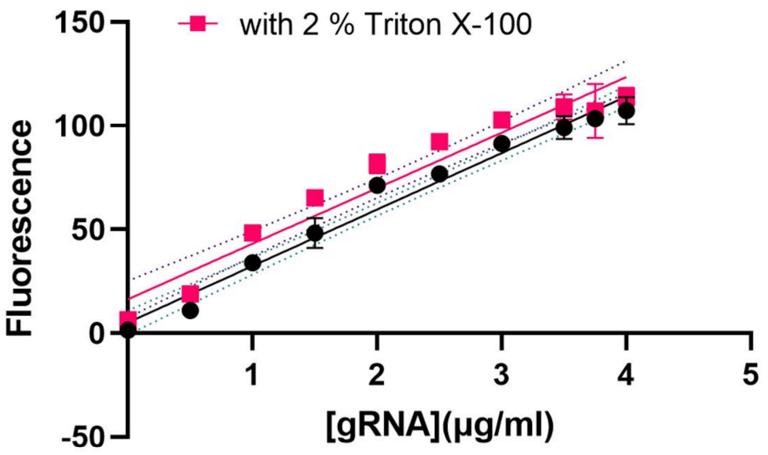

B

	LNP-RNP [HEPES]		LNP-RNP [H_2O]	
	Without 2% triton	With 2% triton	Without 2% triton	With 2% triton
[gRNA](µg/ml)	2.08	3.03	1.93	3.14
Complexation efficiency	**68.6 %**		**63.06 %**	

Figure S8.: Determination of the complexation efficiency of sgRNA in lipid nanoparticles. (**A**) Calibration curve of the fluorescent signal of Quant-iT™ RiboGreen® RNA reagent dependent on gRNA concentration (excitation: 485 nm; emission: 530 nm). Linear fit equation: without 2% Triton X-100 $y = 27.24 \times x + 5.109$ ($R^2 = 0.972$); with 2% Triton X-100 $y = 26.78 \times x + 16.32$ ($R^2 = 0.936$) (**B**) Overview of the gRNA concentrations calculated with the linear fit equation in LNP-RNP samples. Two different RNP conditions were compared (50 mM HEPES pH 7.35 buffer and nuclease-free water). Complexation efficiency was determined by dividing LNP samples treated without 2% Triton X-100 with LNP samples treated with 2% Triton X-100.

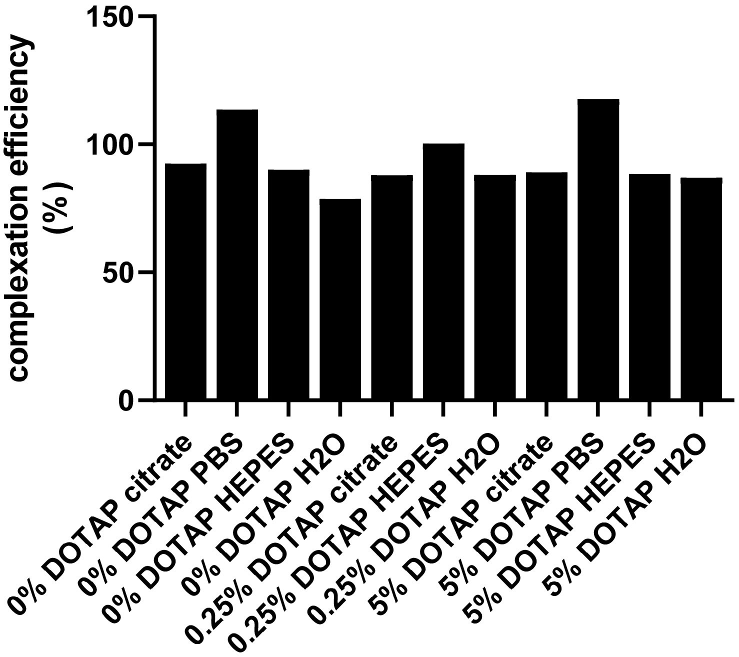

Figure S9.: ***Complexation efficiency of Cas9 to LNPs in different formulation conditions.*** Complexation efficiency was determined by HPLC as shown in figure S.5.1. For each formulation condition, a LNP-RNP was formulated and run on Xbridge protein BEH C4 300Å column as a non-dialyzed and dialyzed sample (to remove free SpCas9). Efficiencies were calculated by dividing the concentration of SpCas9 of the dialyzed samples by the non-dialyzed sample. Concentrations of SpCas9 were determined via EMPOWER software based on standard samples of a calibration curve as depicted in figure S.5.1.c.

Additional information on LNP-RNP tested on stoplight HEK293T cells

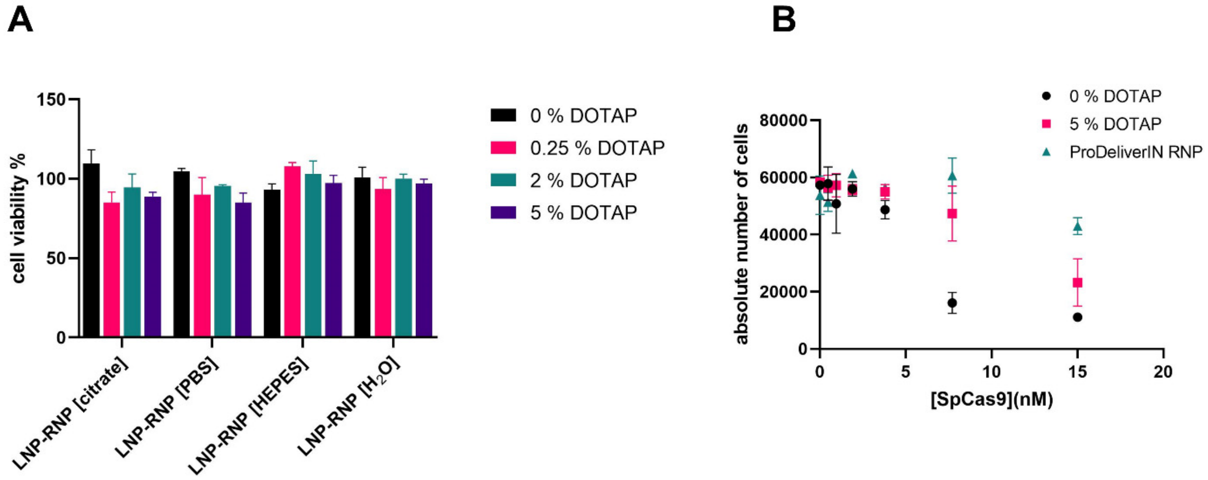

Figure S10.: (**A**) Cytotoxicity assay to determine cell viability of HEK293T stoplight cells after treatment with lipid nanoparticles with final RNP concentration of 7.7 nM in triplicate. Different LNP-RNP samples with different RNP conditions and molar ratio of DOTAP do not show an effect on cell viability. (**B**) Absolute number of stoplight HEK293T cells per well to show cell viability in dependency of dose of LNP-RNP formulations with 0 or 5 mole% DOTAP. Cells were treated in duplicate with nanoparticles. Absolute number of cells treated with LNP were compared to cells treated with commercial transfection agent ProDeliverIN.

Flow cytometry to determine gene knock-out efficiencies in HEK293T stoplight cells

To support the image analysis, gene editing efficiency was also determined by flow cytometry using the BD FACS CANTO II (Becton Dickinson, Franklin Lakes, USA). Cells were harvested off of the Greiner 96-well black plate by trypsinization and transferred to a BD Falcon U-bottom 96 well plate (Becton Dickinson, Franklin Lakes, USA), where the cells were pelleted and washed 2× with 200 µL PBS by centrifugation at 300×*g* for 5 min. The cells were then resuspended and fixed in 1% paraformaldehyde. EGFP fluorescence was measured in the FITC channel, mCherry fluorescence was measured in the PERCP-Cy5 channel. Gene editing efficiency was determined by calculating the parent percentage of EGFP-positive cells in the mCherry-positive cell population using FlowLogic software. Flow cytometry data analysis is represented in figure S.15. and the results are given in figure S.16.

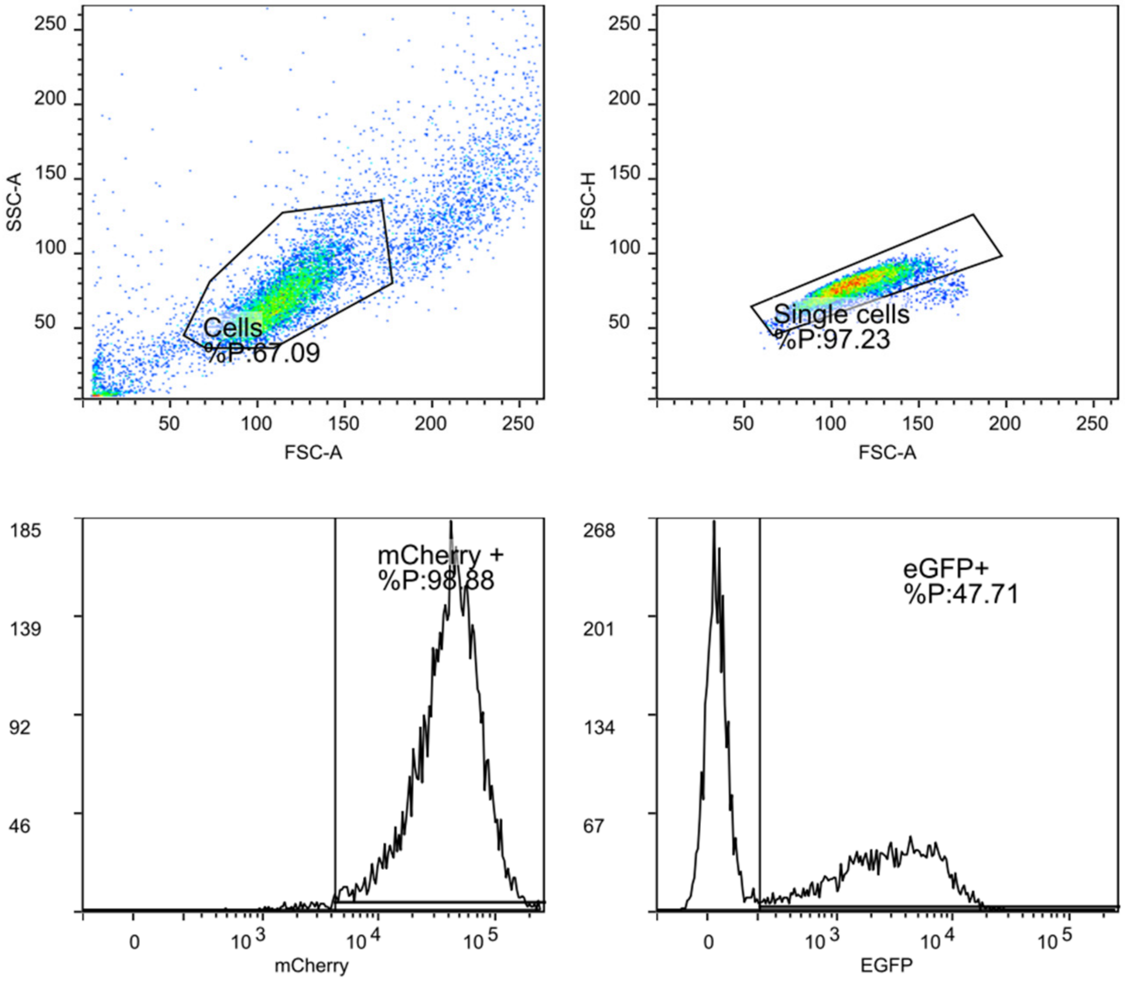

Figure S11.: Top panel shows the gating strategy to determine single cells within the HEK293T stoplight cells during flow cytometry studies. Bottom panel shows selection of mCherry population and within that population the EGFP positive selection.

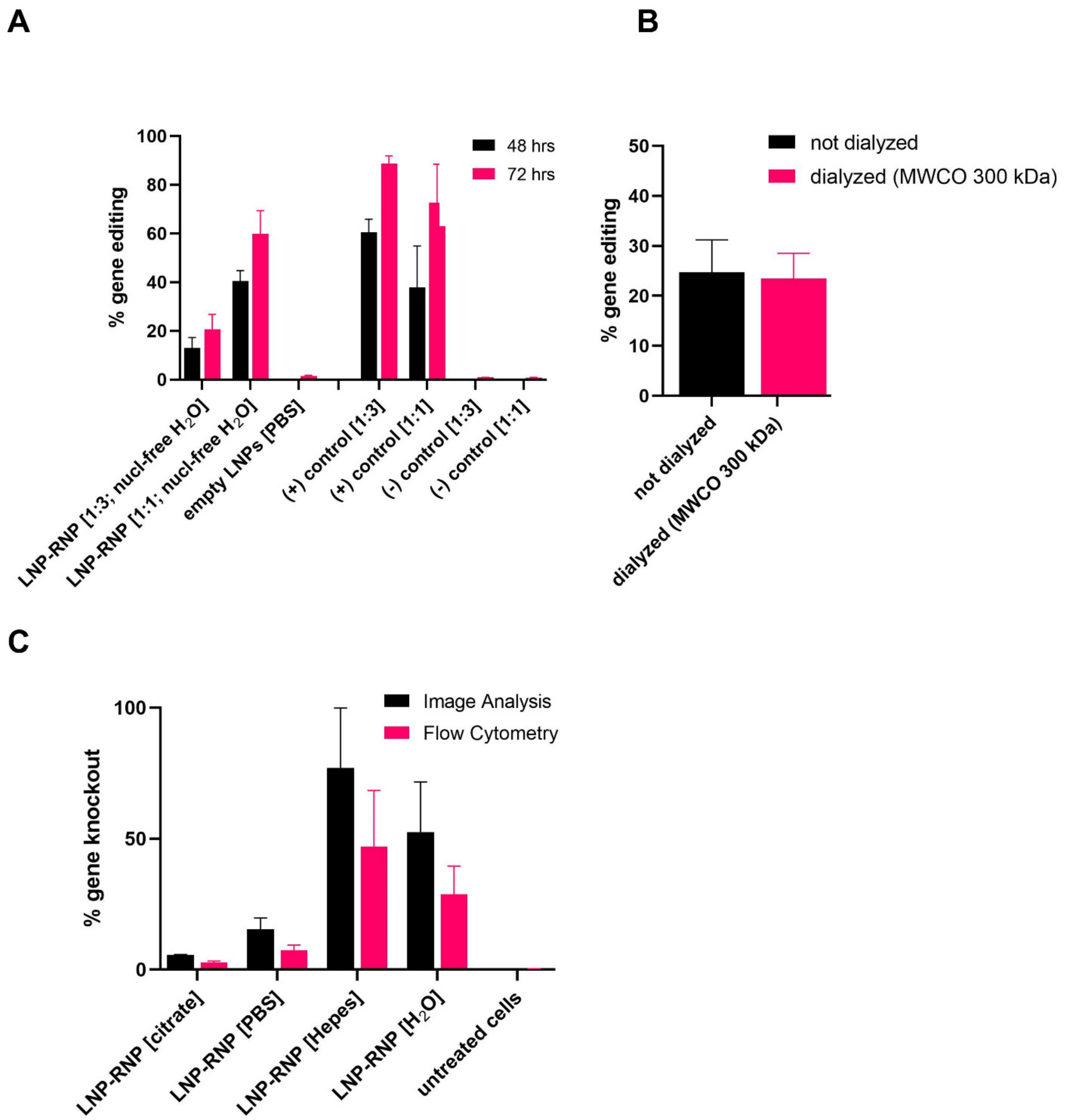

Figure S12.: Optimization of formulation. **(A)** Optimization of molar ratio between SpCas9 protein and gRNA. Gene editing is depicted after 48 h and 72 h of treatment of HEK293T stoplight reporter cells with LNP-RNP. The commercial transfection agent RNAiMAX was used as a positive control (following manufacturer's protocol). As a negative control free RNP at same concentration was added to cells. **(B)** Dialysis of LNP-RNP against 1x HBS with Float-A-Lyzer MWCO 300 kDa does not result in less gene editing than undialyzed LNP formulation. (**C**) Comparison of image analysis and flow cytometry to determine the gene knock-out efficiency of various LNP-RNP formulations. For simplicity, only 5% DOTAP was depicted in this graph. That flow cytometry analysis yields lower gene editing values than image analysis was seen for each complexation condition for RNP and LNP, but the trends follow the same pattern.

Tracking of Indels by Decomposition (TIDE) analysis

TIDE was performed as described by Brinkman et al (S17B) [2]. In short, genomic DNA was isolated 48h after transfection from HEK293T Stoplight cells using the PureLink Genomic DNA Mini Kit (Thermo Fisher, Landsmeer, the Netherlands) following the manufacturer's instructions. The target region was amplified by PCR, using the sequences given in the supplementary information (S2). The PCR product was purified using QIAquick PCR Purification kit (Qiagen GmbH, Hilden, Germany) and Sanger-sequenced. The forward Sanger sequence chromatogram was used for TIDE analysis, by using the TIDE webtool (http://tide.nki.nl). To determine gene modification frequencies, the sequence chromatogram from untreated cells was used as a reference sequence. The percentage of gene editing was calculated with the indel size range set at 25 and the decomposition window fixed between 300–600 bp. The TIDE analysis outputs are provided in Supplementary Figures S14-16.

2. Brinkman, E.K.; Chen, T.; Amendola, M.; van Steensel, B. Easy Quantitative Assessment of Genome Editing by Sequence Trace Decomposition. *Nucleic acids research* **2014**, *42*, e168, doi:10.1093/nar/gku936.

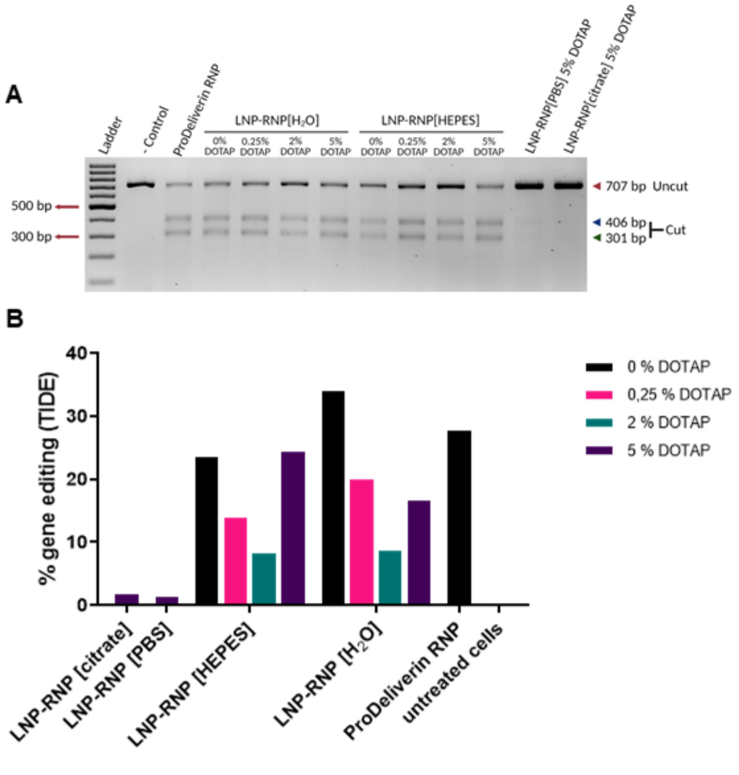

Figure S13.: Genetic readouts of gene-editing in the HEK293T-stoplight cells. (**A**): Original gel of figure 3E. (**B**): Percentage of gene-edited cells found in TIDE analysis performed on the same samples (**n** = 3). These are in line with the functional data provided in figure 3A and 3C.

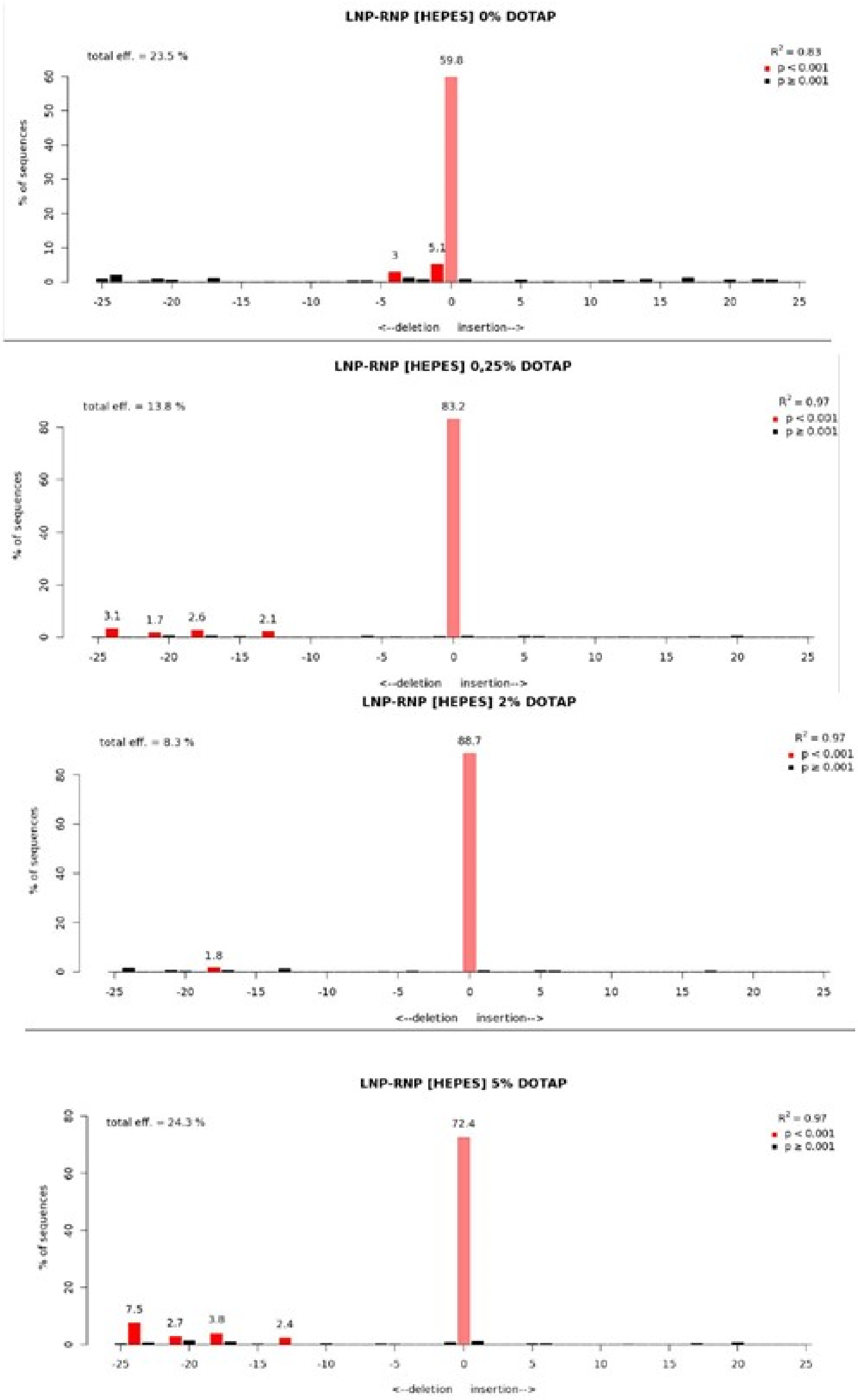

Figure S14. Raw TIDE data for formulations LNP-RNP complexed in 50 mM HEPES buffer pH 7.35 with DOTAP 0, 0.25, 2 and 5 mole%.

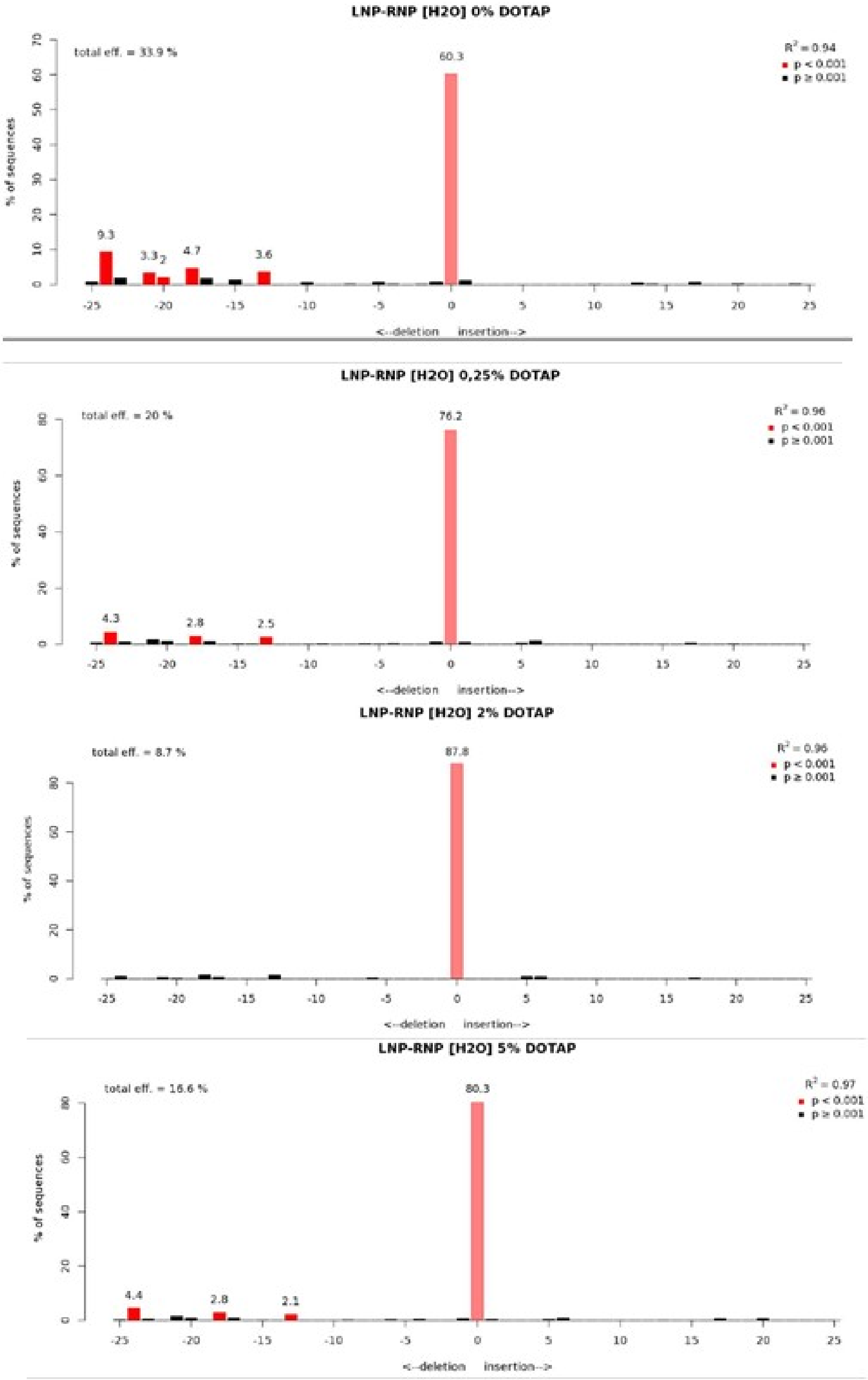

Figure S15. Raw TIDE data for formulations LNP-RNP complexed in nuclease-free water with DOTAP 0, 0.25, 2 and 5 mole%.

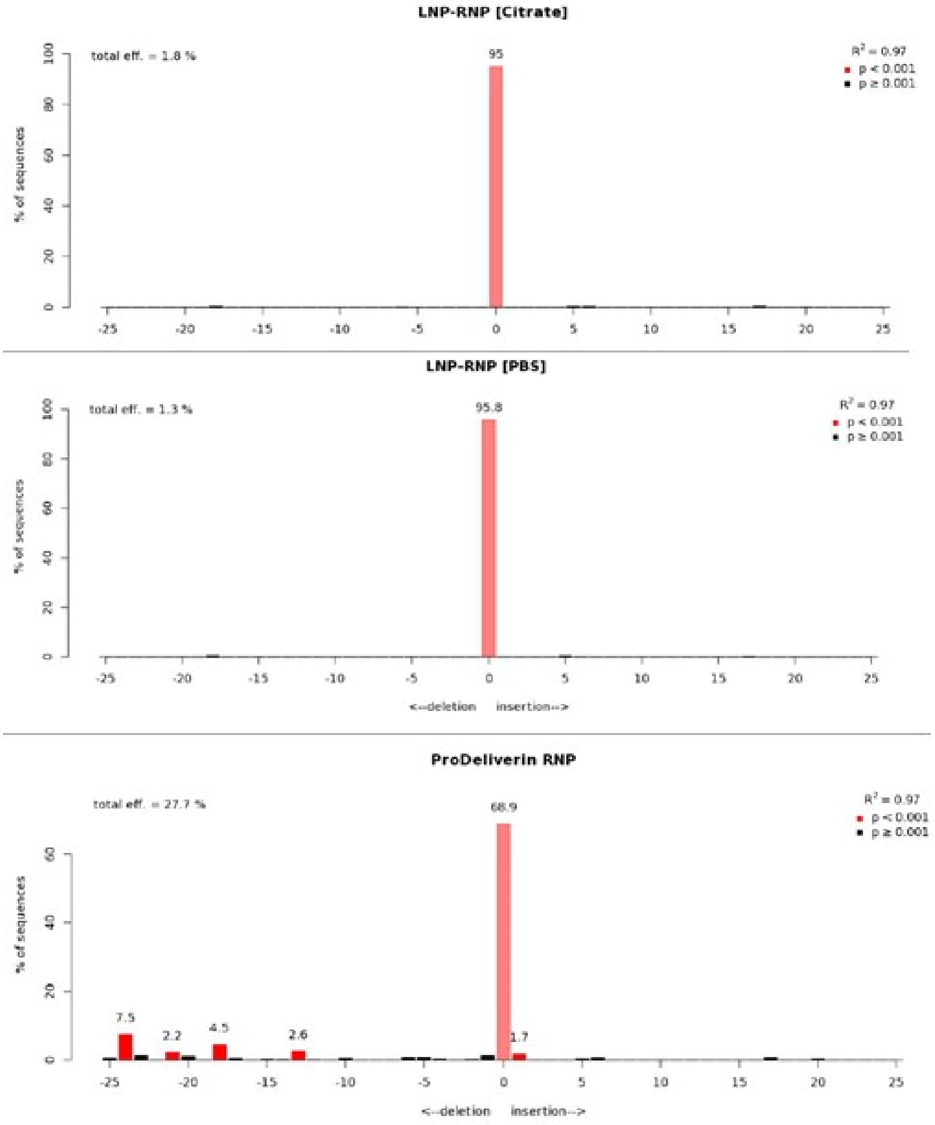

Figure S16. Raw TIDE data for LNP-RNP formulations complexed in citrate (top) and PBS (middle) with DOTAP 5 mole%. Bottom graph is the raw TIDE data for the positive transfection control, ProDeliverIn RNP.

Method statistical analysis

To determine the significant effect of formulation condition, molar ratio of DOTAP, or experimental variation on gene editing outcome a three-way ANOVA was performed on R. To illustrate the experimentally observed determinants of editing efficiency, a recursive partitioning and regression tree was generated using the R-package rpart with the minsplit-parameter (minimum number of observations per node to be considered for splitting) set to 10, otherwise default settings were used. Efficiency was regressed based on the parameters: dotap (0%, 0.25%, 2%, 5%), condition (H20 vs. HEPES), and experimental series (E1, E2 or E3). The generated tree was drawn using the R-package partykit (2).

1) Terry Therneau, Beth Atkinson and Brian Ripley (2017). rpart: Recursive Partitioning and Regression Trees. R package version 4.1-11. https://CRAN.R-project.org/package=rpart

2) Torsten Hothorn, Achim Zeileis (2015). partykit: A Modular Toolkit for Recursive Partytioning in R. Journal of Machine Learning Research, 16, 3905-3909. URL http://jmlr.org/papers/v16/hothorn15a.html

Analysis of results of statistical analysis

Statistical analysis shows that nuclease-free water, especially with lower molar ratio of DOTAP, results in higher gene editing outcomes than particles formulated in HEPES buffer. Formulations made in HEPES buffer seem to require a higher molar ratio of DOTAP.

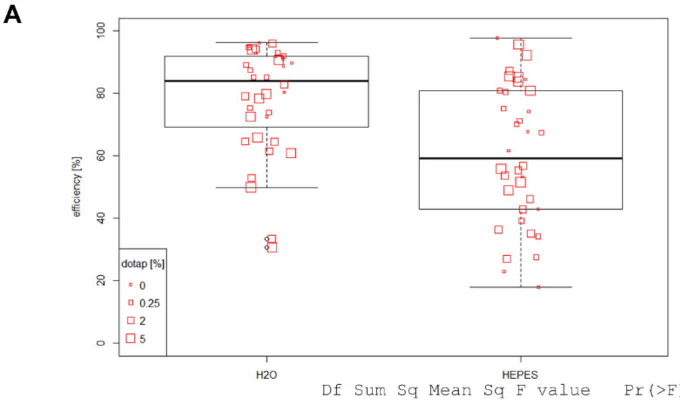


```
                       Df Sum Sq Mean Sq F value   Pr(>F)
df$cond                 1   5642    5642  23.000 1.11e-05 ***
df$exp                  2   2010    1005   4.098 0.021471 *
df$dotap                1     41      41   0.168 0.683385
df$cond:df$exp          2   5134    2567  10.465 0.000126 ***
df$cond:df$dotap        1   3516    3516  14.334 0.000356 ***
df$exp:df$dotap         2   2388    1194   4.868 0.010988 *
df$cond:df$exp:df$dotap 2    129      65   0.263 0.769262
Residuals              60  14717     245
---
Signif. codes:  0 '***' 0.001 '**' 0.01 '*' 0.05 '.' 0.1 ' ' 1
  Tukey multiple comparisons of means
    95% family-wise confidence level
```

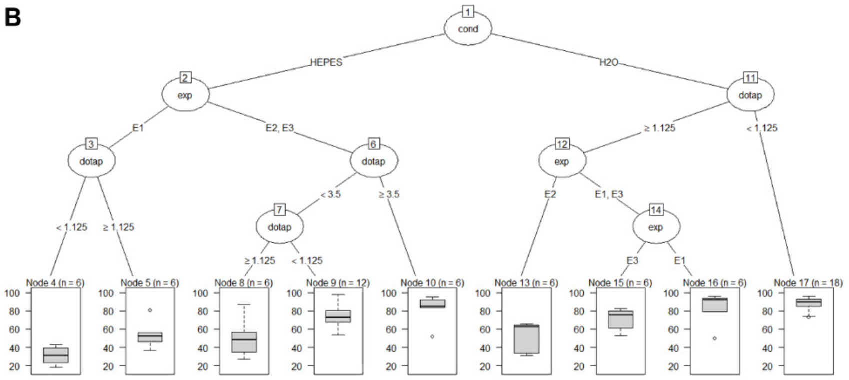

Figure S17.: Statistical analysis to determine the effect of formulation condition, experimental repeat, and molar ratio of DOTAP on gene knock-out efficiency. (**A**) Boxplots show range of gene knockout efficiency on HEK293T stoplight cells for the two formulation conditions 50 mM HEPES buffer pH 7.35 and nuclease-free water (H_2O) over the span of three individual experiments and various formulations with different molar ratios of DOTAP (0%, 0.25%, 2%, 5%). The latter is represented with varying square point sizes (smallest – 0% DOTAP, largest square – 5% DOTAP). Statistical significance is indicated with (*). Cond – HEPES buffer or nuclease-free water; exp – three repeats of experiment, dotap – molar ratio of DOTAP in lipid formulation. (**B**) Recursive partitioning and regression tree to visualize effect of formulation condition, molar ratio of DOTAP, and experimental repeats on gene editing efficiency.

Input Image	**Stack Processing :** Individual Planes<br>**Flatfield Correction :** None		
Find Nuclei	**Channel :** BP445/45<br>**ROI :** None	**Method :** B<br>Common Threshold : 0.4<br>Area : > 30 µm²<br>Split Factor : 7.0<br>Individual Threshold : 0.4<br>Contrast : > 0.1	Output Population : Nuclei
Select Cell Region	**Population :** Nuclei	**Method :** Resize Region [%]<br>Region Type : Nucleus Region<br>Outer Border : -60 %<br>Inner Border : 100 %	Output Region : Nucleus Region
Calculate Intensity Properties	**Channel :** BP600/37<br>**Population :** Nuclei<br>**Region :** Nucleus Region	**Method :** Standard<br>Mean	Output Properties : Intensity Nucleus Region BP600/37
Select Population	**Population :** Nuclei	**Method :** Filter by Property<br>Intensity Nucleus Region BP600/37 Mean : > 250	Output Population : mCherry +
Calculate Intensity Properties (2)	**Channel :** BP525/50<br>**Population :** mCherry +<br>**Region :** Nucleus Region	**Method :** Standard<br>Mean	Output Properties : Intensity Nucleus Region BP525/50
Select Population (2)	**Population :** mCherry +	**Method :** Filter by Property<br>Intensity Nucleus Region BP525/50 Mean : > 200	Output Population : eGFP +
Define Results	**Method :** List of Outputs<br>**Population : mCherry +**<br>Number of Objects<br>Apply to All :<br>Intensity Nucleus Region BP600/37 Mean :<br>Intensity Nucleus Region BP525/50 Mean :<br>eGFP + :<br><br>**Population : Nuclei**<br>Number of Objects<br>Apply to All :<br>Intensity Nucleus Region BP600/37 Mean :<br>mCherry + :<br><br>**Population : eGFP +**<br>Number of Objects<br>Apply to All :<br>Intensity Nucleus Region BP600/37 Mean :<br>Intensity Nucleus Region BP525/50 Mean :<br><br>**Population : mCherry + :** None<br>Population : Nuclei : None<br>Population : eGFP + : None		

Figure S18. Columbus analysis method used to calculate the EGFP-positive population in the Stoplight gene-editing assay.

Supplementary characterization and AF4 data

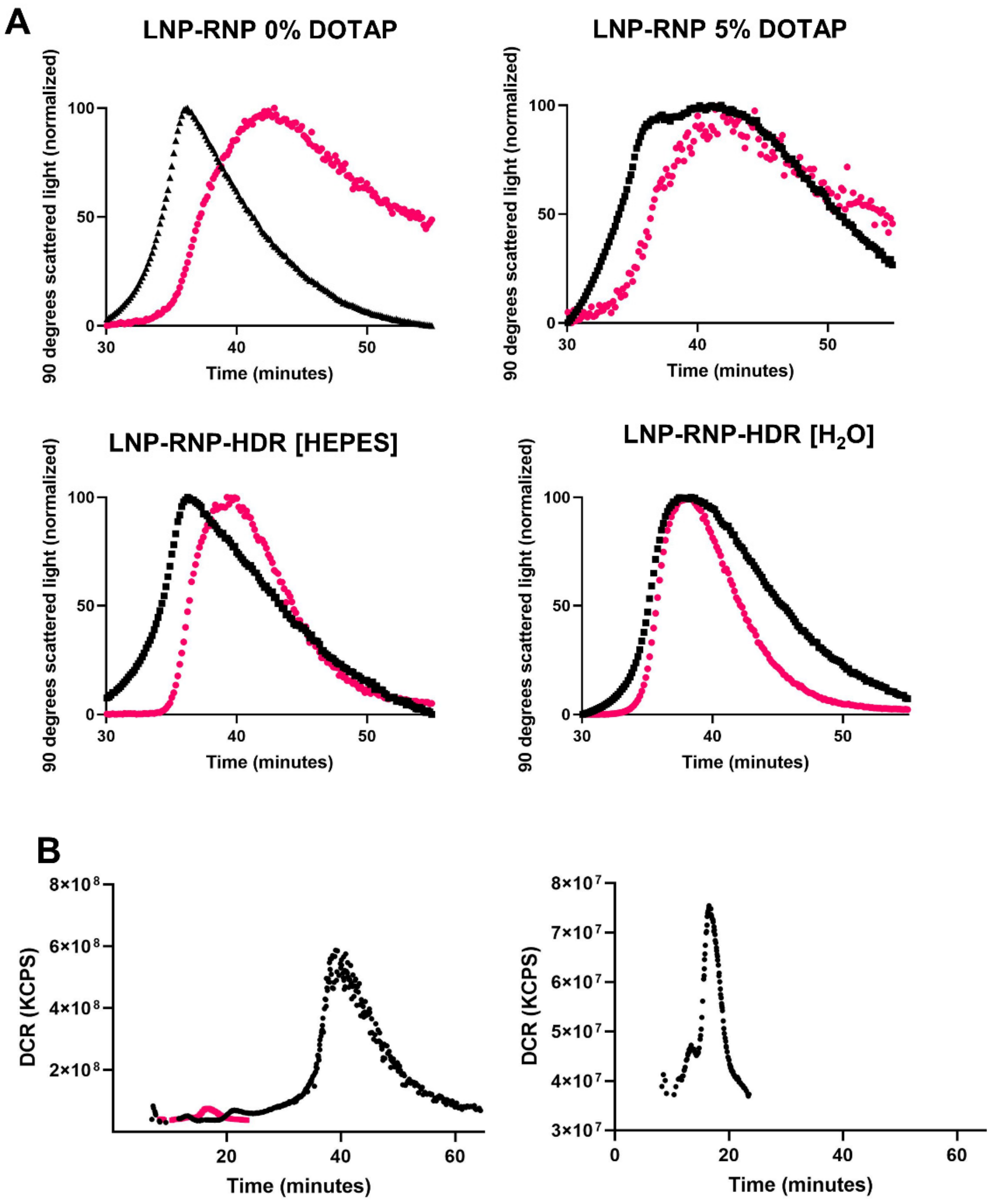

Figure S19.: (**A**): AF4 fractogram of 90 degrees scattered light (normalized) (lowest signal value corresponds to 0% and highest value corresponds to 100 %) to visualize retention time of particles with (black) and without incubation with 20 % plasma (pink). (**B**): Left panel: Overlay fractogram recorded by DLS detector of LNP-RNP-HDR [H2O] particle incubated with plasma (black) and plasma control (pink). Right panel: MALS fractogram recorded by DLS detector of plasma control sample.

Supplementary information on LNP-RNP-HDR formulations

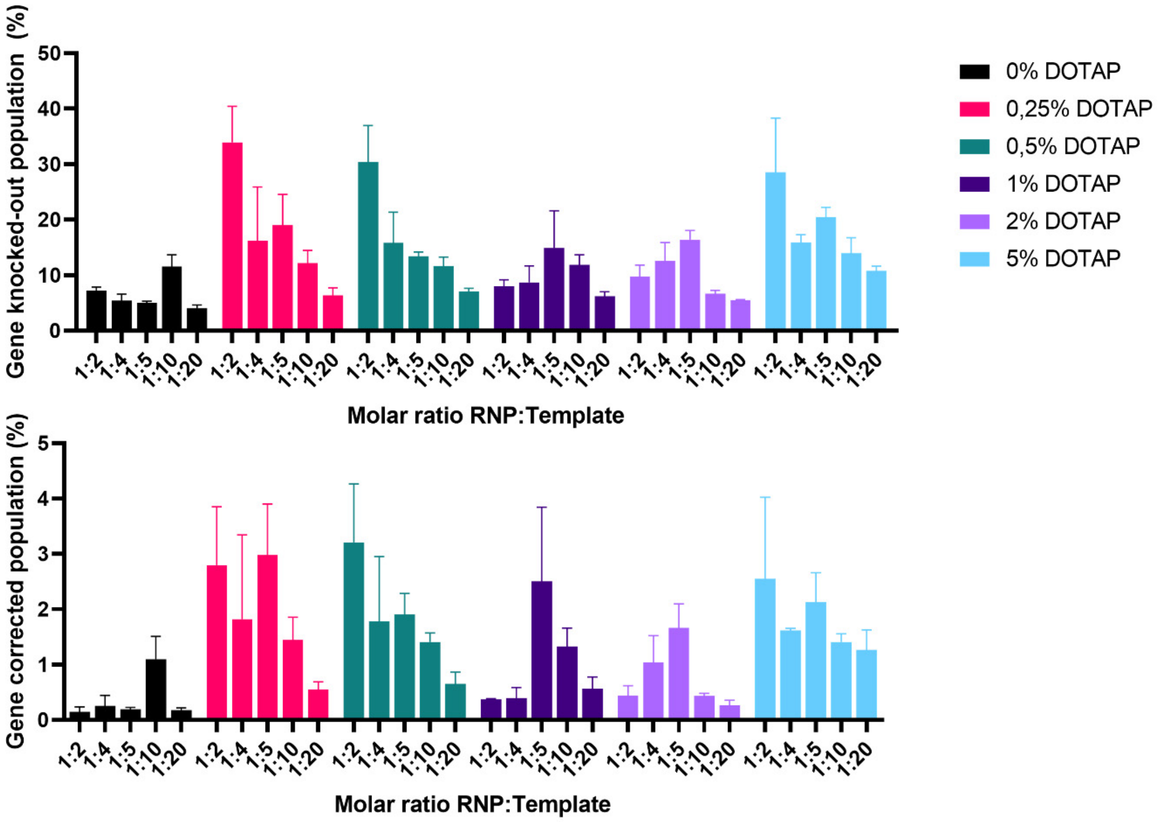

Figure S20. LNP-RNP-HDR [HEPES] optimization study with additional DOTAP concentrations. Removal of DOTAP from the formulation leads to an overall decrease of the editing efficiency.

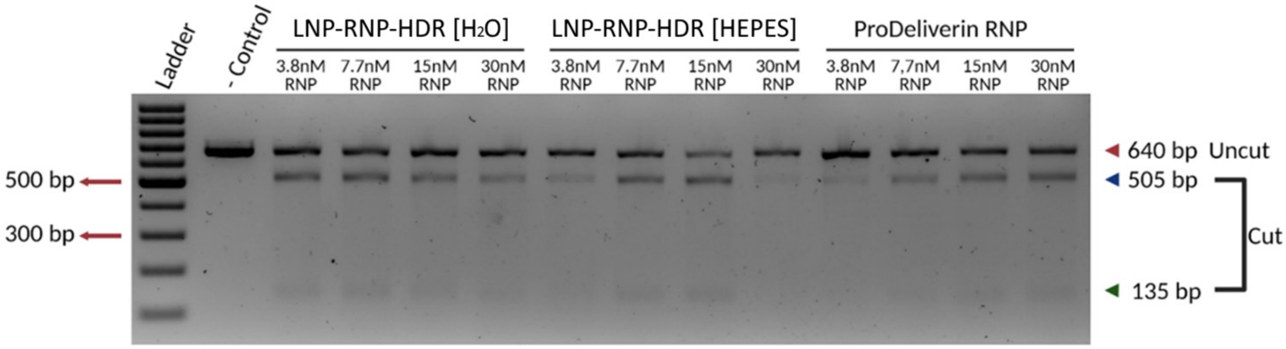

Figure S21.: Full T7E1 assay performed on HEK-EGFP treated with ascending dosages of LNP-RNP-HDR with a 1:2 molar ratio of RNP:HDR template and 0.25% DOTAP in the formulation. ProDeliverIN RNP were prepared with an additional 1:1 molar ratio of HDR template. Cells were harvested from the same population as the flow cytometry data presented in Fig. 6D.
